# E-Library of Authentic Patient Videos Improves Medical Students’ Mental Status Examination

**DOI:** 10.1007/s40596-019-01130-x

**Published:** 2019-11-13

**Authors:** Jessica R. Hansen, Maria Gefke, Ralf Hemmingsen, Cecilie Fog-Petersen, Erica B. Høegh, August Wang, Sidse Marie Arnfred

**Affiliations:** 1 Psychiatry West, Slagelse, Region Zealand Mental Health Service, Slagelse, Denmark; 2 Department of Clinical Medicine, Faculty of Health and Medical Sciences, Copenhagen University, Copenhagen, Denmark; 3 Mental Health Center Amager, Capital Region Mental Health Services, Copenhagen, Denmark

**Keywords:** Clinical decision-making, Blended learning, Psychiatry, Digital, Clerkship

## Abstract

**Objective:**

During psychiatric rotation, clerkship students must learn the clinical skill of recording an accurate Mental Status Examination (MSE). The authors built a video e-library consisting of 23 authentic patient videos that were accessible on a secure website during the rotation period, aimed at assisting students’ acquisition of MSE skills.

**Methods:**

The authors conducted a prospective case comparison study investigating the impact of the video e-library as “add-on” intervention, on acquisition of MSE skills, as measured by a test consisting of three videos with adjoining forced choice questionnaires. Eighty-five clerkship students had instructions and access to the video e-library whereas 82 did not. A group of clinicians, unfamiliar with the video e-library, was also subjected to the new MSE skills test and they served as a reference group. Outcome was defined as scores of MSE skills measured by the purpose made MSE skills test and entailed evaluation questions on the students’ use of the e-library.

**Results:**

The MSE skill test score differed between the three groups, and the clinicians scored higher than both student groups (clinicians mean score (M) 12.6; *p* < 0.001). However, the students with video access scored higher compared to students without access (M 10.7 versus M 9.9, *p* = 0.04). The e-library was appreciated by the students as helpful (83.6%) and they used it not only for practicing the MSE but also for observation of interviewing techniques.

**Conclusion:**

The e-library with video vignettes of authentic patients strengthens MSE skills as “add-on” to the psychiatric rotation, and evaluations by the students were positive.

The clinical skill of recording an accurate Mental Status Examination (MSE) is one of the key elements the clerkship student must learn during psychiatric rotation. At Copenhagen University (CPHU), we developed an e-library comprised by video vignettes of short and authentic patient interviews, as a supplement to the teaching during the clinical rotation in psychiatry [1–4]. The main purpose of the e-library was to help the students practice their MSE skills. The MSE is a significant element in psychiatric assessment and diagnosis [5] and an important tool used in medical records to aid communication among clinicians [6]. Hence, it is all the more important to ensure a consistent teaching/learning of MSE.

After an initial developmental phase, we considered it pertinent to investigate whether access to the video e-library as add-on to standard psychiatric rotation enhances clerkship students’ MSE skills as measured by a forced choice test (MSE skills test). For the MSE skills test, we also included a reference group of clinicians who had no experience with the video e-library. Our hypotheses were that the students with access to the video e-library would score higher on the MSE skills test than those without access, and that clinicians would perform better than both student groups.

## Methods

This study was approved by the University of Copenhagen Ethics Committee for Health and Science and by the Danish Data Security Agency. The patients made informed written consent to the participation in educational videos and we took measures to ensure the protection of personal data by removing auditory, traceable information (surname etc.) from the video vignettes. The enrolled students also signed a study consent form and a confidentiality agreement. All videos and data were stored on a secure server.

Clerkship students, enrolled at CPHU and assigned to a 3-week clinical rotation in psychiatry in their 5th year of the 6-year medical curriculum, were asked to participate in the study. They had rotation in the public psychiatric hospitals of two different geographical regions, with 3 rotation schedules each term, during the fall term of 2017 and the spring term of 2018. Regional allocation of students was carried out *at random* by the faculty office. All students answered baseline questions about their interest in psychiatry (“How interested are you in the psychiatric discipline?” Response options (RO): 1 = very little interest; 2 = little interest; 3 = indifferent/do not know; 4 = strong interest; 5 = very strong interest) and psychiatric clinical experience (“How much clinical psychiatric experience do you have?” RO: 1 = none; 2 = little (i.e., occasionally health care worker in mental health service); 3 = some (i.e., frequently health care worker in mental health service or occasionally apprentice doctor); 4 = much (frequently apprentice doctor); 5 = very much (i.e., apprentice doctor for 3 months). Students in one of the regions received the add-on educational intervention consisting of (1) personal login to the video e-library platform from hospital computers, (2) instructions in use of the e-library, and (3) demonstrations of two video cases and the adjoining MSE texts. The video e-library contained 23 short (8–12 min) video vignettes with authentic patients interviewed purposely for the training of MSE. The study instructions were to watch at least three videos during the rotation, but otherwise, it was a self-study tool at their own convenience.

Students with access to the video e-library also filled out a short evaluation questionnaire concerning the video e-library, herein including the experienced helpfulness (“To what extent did the videos help you learn the MSE?” RO: 1 = not at all; 2 = rather small; 3 = some; 4 = rather much; 5 = very much), the satisfaction with the technical function of the e-library (“Did the video e-library function satisfactorily?” RO: 1 = very unsatisfactorily; 2 = unsatisfactorily; 3 = Adequate; 4 = satisfactorily; 5 = very satisfactorily), the number of videos they had seen (“How many videos did you see on your own?” RO: 1 = none; 2 = 1–2; 3 = 3–6; 4 = 7–12; 5 = more than 12), and what they used the e-library for (“What learning did the you use the video e-library for?” with the option to select up till three of 6 RO: 1 = recognizing phenomena/symptoms/behavior of patients; 2 = using correct terminology; 3 = structuring MSE; 4 = elaborating questions; 5 = nothing; 6 = other).

The MSE skills test was only offered to students participating in this study and not part of routine assessment. It was administered at the end of the 3-week rotation and it consisted of three subtests each consisting of a video vignette, not accessible in the library (i.e., none of the students had seen it previously) with a forced choice test (FCT). A short text on the medical history of the patient initiated the video. Each FCT consisted of 20 descriptive one-sentence statements that could be part of a MSE. The instructions were to mark the five correct statements that entailed a clinically important and precise description of the case at hand. The five correct statements each yielded a score of + 3 points. Five statements were detrimentally wrong, they each yielded a score of − 3 points. The last ten statements were not to the point; they each yielded a score of 0 points. Hence, each FCT subtest ranged from the lowest − 15 points (if only the detrimentally wrong statements were selected) to + 15 points (if only the correct statements were selected). The MSE skills test score consisted of each student’s average score of the three FCTs. Additionally, clinicians attending a 1-day seminar on psychopathology were asked to sit in on an identical MSE skills test as the student groups. We examined MSE skills differences between the three groups (the two student groups and the clinicians) by one-way analyses of variance (ANOVA). The categorical response options in the questionnaires were analyzed as continuous measures and these as well as student baseline variables were tested for difference by Student’s independent two-tailed *t* test.

## Results

**Fig. 1:**
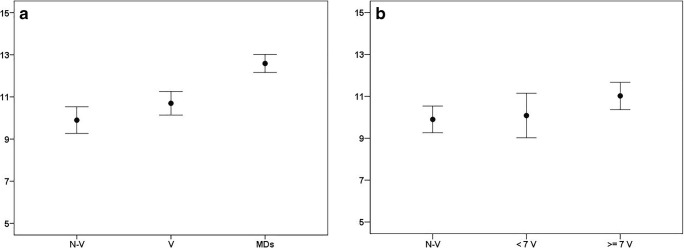
Mental Status Examination Skills Test. **a** Average MSE skills test score. *N*-*V* = students without access to videos, *N* = 59; *V* = students with access to videos, *N* = 69; MDs = reference group of clinicians, *N* = 57. **b** Average MSE skills test score in the student groups, where the students with video access is sub-divided by the number of videos watched, i.e., less than seven videos (< 7V, *N* = 24) or seven or more videos (≥ 7, *N* = 45)

In total, 167 students were included at the beginning of the psychiatric rotation and were expected to participate in the MSE skills testing at the end of the study. By way of drop-out, 128 students attended the test. In all 85 students having video e-library access were included and 69 tested, i.e., a dropout of 19%. Eighty-two students without access were included and 59 tested, i.e., a dropout of 28%. The drop-out difference was not significant. Student groups did not differ in age (mean (M) 26.3 years), gender distribution (females 70%), regarding previous psychiatric experience, which was limited (M 2.1; SD 0.6), or regarding interest in psychiatry, which was moderate (M 3.0; SD 1.40). The clinicians participating in the MSE skills test were 30 psychiatrists and 27 psychiatric residents. MSE skills test score range was 2–15 across the overall student sample (*n* = 128). The score differences between the three groups were highly significant (*p* < 0.001), with the group of clinicians scoring higher than both student groups (M 12.6; standard deviation (SD) 1.6; *p* < 0.001). The students with video e-library access (M 10.7; SD 2.3) scored significantly higher than the students without access (M 9.9; SD 2.4; *p* = 0.04) (see Fig. 1, panel a).

Ninety-nine percent of the students with access to the e-library reported having watched more than two videos, and 64% reported having watched seven or more videos. Exploring whether the number of videos watched affected the test score, we stratified the student group with access to the video e-library according to the median number of videos watched (i.e., seven videos). Hence, when we compared the resulting two sub-groups (i.e., those having watched seven videos or more (*N* = 45) and those having watched less than seven videos (*N* = 24)) with the student group without access to the videos, the difference was significant (*p* = 0.05). Post hoc analyses only showed a difference between the students who had watched seven or more videos and the students without video access (Students having watched seven or more videos: M 11.0; SD 2.2; *p* = 0.02) (see Fig. 1, panel b). In the student group with video access, 83.6% stated that the videos provided some or much help in learning to conduct an MSE and 77% found the video library to function satisfactorily or very satisfactorily. The majority (91%) of students reported that they used the video e-library to learn to recognize the psychiatric phenomena/symptoms and behavior, 74% used it for MSE structure, and 70 % used it for terminology. Furthermore, 36% of the students reported that they used it for learning how to elaborate on questions.

## Discussion

We have shown an improvement in MSE skills among students that had access to a video e-library during psychiatric rotation in accordance with our study hypothesis. The improvement was particularly seen when students watched more than seven videos. Furthermore, as hypothesized, psychiatrists and psychiatric residents did better on the test than students, thus conferring criterion validity to the test.

The positive training effect of the video library is in line with a study by Pohl et al. comparing 3 methods of teaching MSE: lectures, authentic videos, or live simulations. They reported that students in the authentic video class performed better at a multiple choice test than their counterparts in the lecture class, when comparing post hoc scores for the two teaching methods. No difference was seen between live simulation and video demonstrations [7]. Also, Sturgeon et al. showed video assisted teaching of mental status examination enabled students to more accurately observe mental status during a live interview test when compared to students who received seminar sessions as a method of teaching MSE [8]. The above findings may indicate a benefit of video assisted teaching on observational skills, including MSE, when transferred to clinical encounters. The reason for such benefit, we speculate, could be that the video e-library offers students a setting to really “dig in” and concentrate on the one task of MSE assessment, whereas a clinical encounter can be much more challenging to navigate with many simultaneous tasks including communication and managing the environment.

Several limitations have to be considered. The attrition percentage was relatively high (19 and 28%) and it is possible that students with less confidence in their own MSE abilities would be more likely to drop out. As the standard curriculum-based clinical clerkship included practice of MSE skills by observing and conducting live interviews with patients for both student groups, the effect of low self-confidence would presumably be the same in the two student groups and as such should not impact the observed difference. The students in both groups were informed at inclusion that their participation in the study and the MSE skills test would not in any way influence their grade at the term exam in psychiatry. Considering the study design, the difference in geographical Health Service affiliation between the student groups could be perceived as a confounder. However, the student distribution was random and done by the CPHU administration office. Moreover, it should be noted that the students with access to the e-library did their clerkship in the Health Service with a much lower density of psychiatrists, and despite this deprivation of specialist guidance actually performed better on the MSE skills test, thereby testifying to the teaching potential of the e-library. Furthermore, it could be considered a weakness that the number of videos watched was self-reported by the students. However, digital tracking of video file access was not possible and would not be an objective measure for the actual amount of attention each student paid to the video. Moreover, it was part of the self-study quality of the video e-library that it would depend on perceived benefit and motivation whether students used it a lot or not. Recall might have been wrong, but we see no obvious incentive, as mentioned above, for systematic under- or over-reporting. Finally, we do not know the sensitivity or specificity of our MSE skills test, which was designed for this study, and therefore further validation of the test is necessary. The three patient interviews used in the MSE skills test were picked as they were deemed to be of equal MSE difficulty to the vignettes in the e-library, yet we had no objective measure of this.

In conclusion, a video e-library with short video vignettes of authentic patients strengthens the process of acquiring MSE skills and thereby provide a promising add-on to the clinical rotation. This could particularly be of use in psychiatric hospitals where the number of psychiatrists and faculty per student is low. Future studies must clarify whether a video e-library constitutes a learning advantage as an add-on in any setting, including departments with a high density of specialists.
